# Composition and variation of respiratory microbiota in healthy military personnel

**DOI:** 10.1371/journal.pone.0188461

**Published:** 2017-12-07

**Authors:** Jun Hang, Nela Zavaljevski, Yu Yang, Valmik Desai, Richard C. Ruck, Louis R. Macareo, Richard G. Jarman, Jaques Reifman, Robert A. Kuschner, Paul B. Keiser

**Affiliations:** 1 Viral Diseases Branch, Walter Reed Army Institute of Research, Silver Spring, Maryland, United States of America; 2 Department of Defense Biotechnology High Performance Computing Software Applications Institute, Telemedicine and Advanced Technology Research Center, US Army Medical Research and Materiel Command, Fort Detrick, Maryland, United States of America; Columbia University, UNITED STATES

## Abstract

Certain occupational and geographical exposures have been associated with an increased risk of lung disease. As a baseline for future studies, we sought to characterize the upper respiratory microbiomes of healthy military personnel in a garrison environment. Nasal, oropharyngeal, and nasopharyngeal swabs were collected from 50 healthy active duty volunteers eight times over the course of one year (1107 swabs, completion rate = 92.25%) and subjected to pyrosequencing of the V1–V3 region of 16S rDNA. Respiratory bacterial taxa were characterized at the genus level, using QIIME 1.8 and the Ribosomal Database Project classifier. High levels of *Staphylococcus*, *Corynebacterium*, and *Propionibacterium* were observed among both nasal and nasopharyngeal microbiota, comprising more than 75% of all operational taxonomical units (OTUs). In contrast, *Streptococcus* was the sole dominant bacterial genus (approximately 50% of all OTUs) in the oropharynx. The average bacterial diversity was greater in the oropharynx than in the nasal or nasopharyngeal region at all time points. Diversity analysis indicated a significant overlap between nasal and nasopharyngeal samples, whereas oropharyngeal samples formed a cluster distinct from these two regions. The study produced a large set of pyrosequencing data on the V1–V3 region of bacterial 16S rDNA for the respiratory microbiomes of healthy active duty Service Members. Pre-processing of sequencing reads showed good data quality. The derived microbiome profiles were consistent both internally and with previous reports, suggesting their utility for further analyses and association studies based on sequence and demographic data.

## Introduction

A number of microbiome studies of the human respiratory regions have investigated individuals or populations of different ages, with or without acute or chronic disease [1–7]. The results of these studies suggest the existence of core respiratory microbiome and a possible association between abnormal microbiota and respiratory diseases [8–10]. Military personnel may be exposed to inhalational hazards, which may increase their risk of contracting acute or chronic respiratory diseases [11, 12]. For example, burn pit exposures in the recent conflicts in Iraq and Afghanistan may have long-term health consequences to respiratory illnesses in the upper airways of Service Members. We initiated a study to characterize the upper respiratory microbiomes of healthy military personnel in a garrison environment in order to establish a baseline for future studies on the effects of specific activities and exposures on respiratory microbiota.

We previously conducted a preliminary study of laboratory and data processing/analysis procedures [13]. The methodology, which was established by using the microbial mock communities developed by the Human Microbiome Project (HMP), has been used to obtain results comparable or superior to those of other studies [14]. The workflow was applied to a pilot run of throat swabs collected from eight subjects [13]. Here, we used the same methods to study respiratory microbiota in a larger group of military volunteers. This project aimed to characterize the composition and temporal variation of the normal military respiratory microbiota, identify individuals with abnormal respiratory bacterial taxa, collect comprehensive demographic data, and uncover associations between variables and profiles of respiratory bacterial communities.

## Materials and methods

### Sampling and DNA isolation

The study protocol WRAIR#1914 and protocol amendment were approved by the Walter Reed Army Institute of Research (WRAIR) Institutional Review Board. Written, informed consent was obtained with signature from each participant. One Ultra-thin minitip flocked swab (dry tube, Catalog number 560C; COPAN Diagnostics Inc., Murrieta, CA) was applied to each of three anatomic sites: the anterior nares, oropharynx, and nasopharynx. The side from which the sample was collected was left to volunteer preference or chosen arbitrarily. The swabs were kept in their tubes and stored at -80°C in WRAIR until they were processed for DNA isolation. The method for extracting DNA from the swabs, which was established in a previous study [13], was applied to this study with modifications. The swabs were processed in parallel by using 96-well plates. The swab tips were cut into the wells in a 96 Deepwell plate (Catalog number 0030 131.517; Eppendorf, Hauppauge, NY). A 450-μl aliquot of pre-chilled lysis solution containing 20 mM TrisCl, pH8.0, 2 mM EDTA, 1mM DTT, 1.2% Triton X-100, 1 mg/ml Lysozyme, and 0.1 mg/ml lysostaphin (Sigma, St Louis, MO) was added to each well. The plate was incubated at 37°C for 90 min and mixed by shaking at 1000 rpm for 2 min on an Eppendorf MixMate before and after incubation. Subsequently, 25 μl of 10 mg/ml Proteinase K (Qiagen, Germantown, MD, USA) was added to each well and mixed. A 300-μl aliquot of the mixture was transferred from each well into a new 96 Deepwell plate, after which 300 μl of Qiagen Buffer AL without ethanol was added. The new plate was then mixed at 1000 rpm for 2 min and incubated at 56°C for 2 h. Ethanol (300 μl) was then added and mixed by pipetting 6 times. From each well, 0.9 ml of the mixture was transferred into a well of a DNeasy 96 DNA-binding plate. DNeasy 96 extraction kit and QIAvac 96 vacuum manifold (Qiagen) were used in DNA purification.

### PCR and Roche 454 pyrosequencing

As described previously [13], the purified DNA samples were subjected to 16S rDNA quantitative PCR to determine Ct values, using Taqman Universal Master Mix II (Invitrogen, Carlsbad, CA) with 321F2 (ACTGAGAYACGGYCCA) and 533R (TTACCGCGGCTGCTGGC) as the primers and 338P (FAM-ACTCCTACGGGAGGCAGCAGT-Black Hole Quencher) as the probe. PCR amplification of the V1–V3 region of 16S rDNA was performed by using the published Human Microbiome Project protocol and modified fusion primers LB-27F2 (CCTATCCCCTGTGTGCCTTGGCAGTCTCAG-AGAGTTTGATCMTGGCTCAG) and LARL1-533R (CCATCTCATCCCTGCGTGTCTCCGACTCAG-ACACGACGACTTTACCGCGGCTGCTGGC), which were shown to improve amplification of some bacteria [13]. PCR cycle number of 20, 25, or 30 was chosen for each sample depending on its Ct value from the 16S qPCR assay, as described previously [13]. Pyrosequencing of the 16S V1–V3 region was performed by using a Roche 454 GS FLX+ system and reagents (Roche 454 Life Sciences Corporation, Branford, CT). Genomic DNA from Microbial Mock Community A, Even, Low Concentration (HM-278D v3.1, BEI Resources) was diluted 10 times and used as a positive control for PCR and pyrosequencing of 16S amplicons.

### Pyrosequencing data processing and taxonomic classification

Sequencing data analysis was based on the Quantitative Insights Into Microbial Ecology (QIIME) pipeline version 1.8 [15]; details of the workflow implementation have been described previously. After demultiplexing sff files, raw reads were filtered to ensure read quality by using the following steps: terminal trimming to remove N from the 3′-end of the raw reads; removal of reads smaller than 200 bases or larger than 1000 bases; removal of reads with a homopolymer eight bases or longer; removal of reads with more than one error in the 16S primer 539R sequence; read trimming to remove primer and linker sequences; and sliding-window trimming with a window width of 50 bases to remove terminal sequences within the window with an average quality score below 25. Chimera filtering was subsequently performed, using the UCHIME algorithm by either the reference-based or *de novo* method [16]. Reads that were classified as chimeric by both methods were removed. Finally, singleton reads were excluded from further analysis. The taxonomic classification of the quality-processed reads was based on the closed reference clustering of sequences into operational taxonomical units, using the UCLUST tool with the sequence identity level at 97%. The read clusters were further assigned to taxonomies, using the RDP classifier with a confidence level of 80% [17]. The microbial profiles obtained after this step contained various hierarchical levels of taxonomy classification and their positions in the taxonomy were used to assess the diversity of each community. In the statistical analyses, reads assigned to taxonomy levels below the genus level were mapped to the corresponding genus level for further evaluation of statistical significance at the genus level.

### Microbiota diversity estimation and statistical analysis

We used the microbiome profiles from QIIME/RDP analysis to evaluate the microbial community diversity within a sample (α-diversity) and the diversity between samples (β-diversity). We estimated α-diversity for all samples by using Shannon entropy measures as implemented in the R-package vegan (https://cran.r-project.org/web/packages/vegan), and used the Wilcoxon signed-rank test [18] implemented in R (http://www.R-project.org/) to compare α-diversities. We analyzed β-diversity by using the weighted UniFrac distance in the QIIME implementation of UniFrac [19] as a measure. The results were visualized by using EMPeror [20].

### Pyrosequencing and data analysis

The resulting 16S V1–V3 amplicon libraries were sequenced by using a Roche 454 GS FLX+ system and reagents (Roche 454 Life Sciences Corporation, Branford, CT, USA). Genomic DNA from Microbial Mock Community A, Even, Low Concentration (HM-278D v3.1, BEI Resources) obtained as part of the Human Microbiome Project [21], was diluted 10 times and used as a positive control for PCR and pyrosequencing of 16S amplicons.

The pyrosequencing data analysis pipeline was based on the Quantitative Insights Into Microbial Ecology (QIIME) pipeline version 1.8 [15]. The 454 read data were subjected to quality processing, chimera filtering, and removal of singleton reads. The taxonomic classification of the quality-processed reads was based on the closed reference clustering of sequences into operational taxonomical units (OTUs), using the UCLUST tool [22] with a sequence identity level of 97%. The read clusters were further assigned to taxonomies by using the Ribosomal Database Project (RDP) classifier [17] with a confidence level of 80%. The bacterial community diversity within a sample (α-diversity) was assessed by using the R-package vegan (https://cran.r-project.org/web/packages/vegan). The diversity between samples (β-diversity) was analyzed by using the QIIME implementation of UniFrac [19] and the weighted UniFrac distance as a measure of β-diversity.

## Results and discussion

### Subject recruitment and characteristics

**Fig 1:**
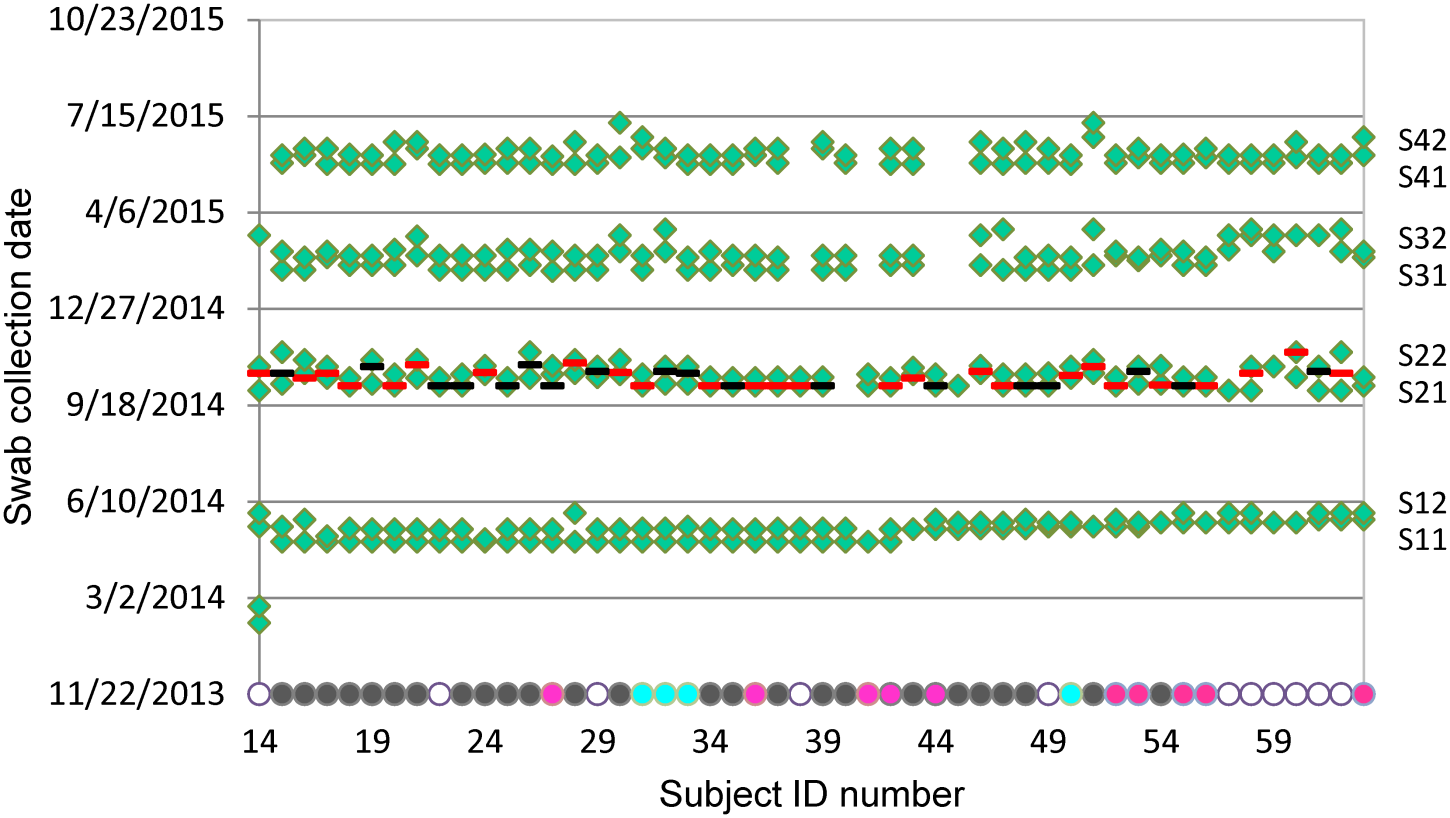
Sample collection from 50 human subjects. Three swabs (nasal, oropharyngeal, and nasopharyngeal) were collected from each volunteer (subject numbers 14–63) during each of two visits (1/2) in every quarter (i.e., S1–S4). *Green diamond* symbols show swab collection dates. *Red bars* denote flu shot vaccination dates (26 subjects). *Black bars* denote Flu Mist vaccination dates (19 subjects). The *dots* on the X-axis filled in with color show musician types: *Empty*, non-musician (11); *Green*, pianist or conductor (2); *Purple*, percussionist (5); *Cyan*, vocalist (4); *Yellow*, violinist (3); *Gray*, wind instrumentalist (25).

A total of 1107 nasal, oropharyngeal, and nasopharyngeal swabs, which represented 92.25% of the total number of planned samples (1200), were collected from 50 volunteers (Table 1, Fig 1). The volunteers were active duty military (39 males and 11 females), including members of the US Army Band and medics from the 3^rd^ US Infantry Regimental Aid Station, Fort Myer, VA. These participants were chosen because they were located near our institute and motivated to participate. In addition, musicians in the US Army Band at Fort Myer (“Pershing’s Own”), unlike most other military personnel, are likely to be serving in the same location for many years and hence available for long-term follow-up studies. Samples were collected at 8 time points over the course of a year (two visits, ideally 1–2 weeks apart, in each of four quarters from mid-2014 to mid-2015) and from 3 anatomical sites: the anterior nares, oropharynx, and nasopharynx (S1 Table). Prior to the influenza season during that year, 26 subjects received the intramuscular influenza vaccine (flu shot), 19 subjects received the intranasal influenza vaccine (Flu Mist), and 5 subjects were not immunized (S1 Table). This made it possible to assess the impact of vaccination route.

**Table 1:** Demographics of the 50 subjects enrolled in the study. The information was summarized based on questionnaires filled in by the subjects during enrollment or each visit. Data on chronic and acute illnesses were also collected from the subjects.

Characteristics	Data
**Gender**	Male (39), female (11)
**Race**	White (43), Hispanic (4), Asian (1), black (1), other (1)
**Occupation**	Medics (7), band musicians (43) Primary instruments include wind (26), percussion (6), vocalist (4), string (3), non-musician staff (4), unknown (3)
**Median age**	37.5
**Median age (medics)**	21, range 20–28
**Median age (band)**	40, range 21–60
**Brushing teeth**	Less than once daily (2), Once daily (17), Twice daily (22), Three times daily or more (9)
**Flossing**	1–3 times a week (18), 4–6 times a week (4), Daily (14), Less than once a week (13), Blank (1)
**Mouthwash**	1–3 times a week (12), 4–6 times a week (4), Daily (12), Less than once a week (19), No (1), Yes with unknown frequency (2)
**Tobacco use**	Smoke (8), chew (2)
**Allergy/Asthma meds**	Oral antihistamine (9),intranasal steroid (5), inhaled beta agonist (4), leukotriene antagonist (2), inhaled steroid (1) used by 13 subjects
**Flu vaccination**	Intramuscular (26), intranasal (19)

### Data acquired and quality assurance

We investigated technical issues related to data quality including primer design, 16S sequences from swab or reagents, and PCR cycle numbers as well as reaction parameters. Performance was evaluated with even and uneven mixes of HMP microbial mock community DNA as well as a mixture of bacterial cells [13]. One of the HMP reference materials, HM-278D, was included in each run of this study for quality control purposes to assure technical consistency across experiments. We used 16S quantitative PCR to identify DNA extracts with exceedingly low 16S rDNA copy numbers (Ct values greater than 30). Although these samples were included in the initial analysis, their abnormal concentrations were taken into account in subsequent data analyses and interpretation of results.

The swabs from same quarter were processed in consecutive days. The samples to be extracted were arranged roughly in the order of their collection time, not grouped by either subject or body site. The three swabs from a same subject and visit were processed in parallel. In total, 18 Roche 454 FLX+ sequencing runs were conducted with the production of total data of 15 Gbases in sequence or 41867 Mbytes in volume. Three thousand or more sequence reads were obtained from each sample (S1 Table), except for those with high Ct values in 16S quantitative assays. Approximately 72% of the raw reads passed quality processing and were used in subsequent analyses. The Roche 454 FLX+ sequencing raw read data (sff files) were deposited in the NCBI Sequence Read Archive database (http://ncbi.nlm.nih.gov/sra) under BioProject PRJNA339931, BioSample SAMN05615462, with 1107 SRA accession numbers listed in S1 Table (https://www.ncbi.nlm.nih.gov/bioproject/?term=PRJNA339931). Sample information and basic statistics of the sequences are summarized in S1 Table.

### Respiratory bacterial community composition and ‘core microbiome’

**Fig 2:**
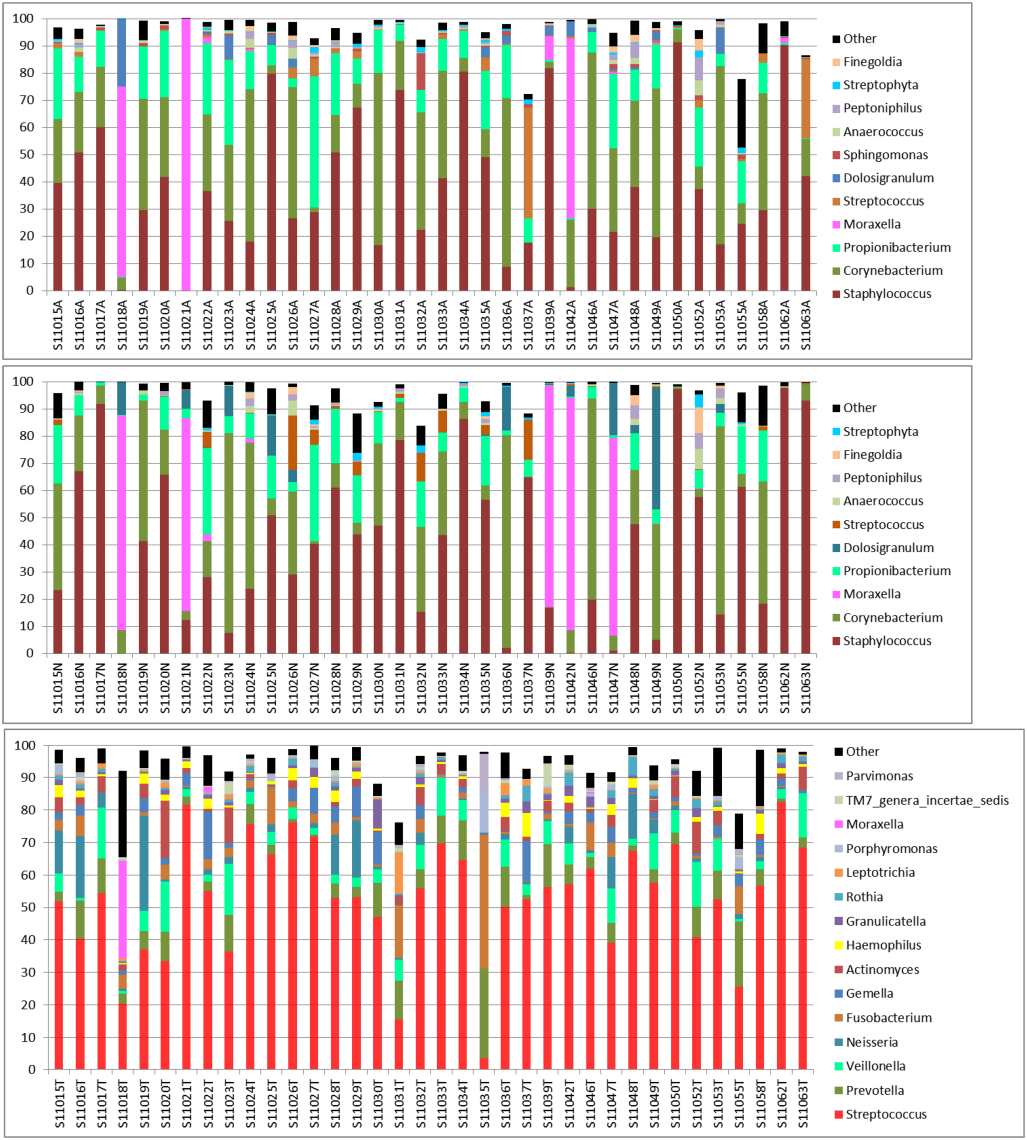
Bacterial community composition of respiratory microbiota in 36 human subjects. Bacterial genera with an average relative abundance of ≥0.5% across the 36 samples are shown. Reads that were classified at the higher taxonomic levels other than genus are denoted as “other”. Each sample name includes the quarter number S1–S4 (*first digit*), visit 1 or 2 (*second digit*), subject number (*last three digits*), and type of swab (A, anterior nares [top panel]; N, nasopharynx [middle panel]; T, oropharynx [bottom panel]).

OTUs clustered at 97% similarity were classified at the genus level by using QIIME 1.8 and the RDP classifier downloaded on July 2015. Fig 2 shows bacterial taxa with an average relative abundance of greater than 0.5% for samples collected on the first visit in each of the four quarters (S11 [i.e., quarter 1 visit 1]; S1 Fig for S11, S21, S31, and S41 [visit 1 in each quarter]). The results for 36 volunteers with samples collected on all eight visits were used in the comparison. Among these, two first-visit samples had high Ct values in 16S quantitative PCR and an insufficient number of sequence reads. Therefore, the corresponding second-visit samples, S22015N and S32017A, were used instead.

The set of major bacterial taxa (genera with a relative abundance of ≥0.5% or ≥1.0%) that exist in >50% of the subjects was defined as the ‘core microbiome’ (Table 2). Both nasal and nasopharyngeal microbiota contained a high content of the same three genera—*Staphylococcus*, *Corynebacterium*, and *Propionibacterium*—which together constituted on average 77.4% and 75.6% of all OTUs classified to genus and OTUs classified to higher levels (shown as ‘other’ on Fig 2), respectively. In contrast, *Streptococcus* was the sole dominant bacterial genus in the oropharyngeal microbiota, although various other bacteria were also present but at much lower levels (Table 2).

**Table 2:** Major bacterial taxa of human respiratory microbiota. Analysis of data (quarter S1 visit 1) from 36 subjects with samples available for all four quarters, showing the percentage of samples with a relative abundance of ≥0.5% or ≥1.0%. Data are shown in bold for percentage of samples ≥50%.

Bacterial genus	Relative abundance (%)		Percentage of samples with abundance	
	Average	Standard deviation	≥ 0.5%	≥ 1.0%
**Anterior nares**				
*Staphylococcus*	38.6	25.4	**94.4**	**94.4**
*Corynebacterium*	25.5	20.8	**91.7**	**91.7**
*Propionibacterium*	13.3	10.8	**91.7**	**80.6**
*Streptococcus*	3.0	8.1	**50.0**	33.3
*Anaerococcus*	0.8	1.2	**52.8**	22.2
**Nasopharynx**				
*Staphylococcus*	42.0	30.2	**94.4**	**94.4**
*Corynebacterium*	22.6	24.0	**88.9**	**86.1**
*Propionibacterium*	11.0	27.4	**83.3**	**83.3**
**Oropharynx**				
*Streptococcus*	52.8	18.3	**100**	**100**
*Prevotella*	7.1	5.7	**100**	**94.4**
*Veillonella*	6.1	4.7	**97.2**	**88.9**
*Neisseria*	4.3	6.7	**77.8**	**58.3**
*Fusobacterium*	4.0	7.3	**80.6**	**55.6**
*Gemella*	3.4	3.5	**86.1**	**72.2**
*Actinomyces*	3.4	3.5	**83.3**	**77.8**
*Haemophilus*	1.9	1.7	**80.6**	**55.6**
*Granulicatella*	1.4	1.6	**72.2**	**52.8**
*Rothia*	1.2	1.3	**66.7**	47.2

Overall, bacterial taxa with an average relative abundance of ≥0.5% constituted over 90% of the respiratory microbiota for all 432 samples (Fig 2; S1 Fig). A few subjects (1 to 5 out of 36 subjects at each collection time point) had taxa with an abundance of >10%, which did not belong to the representative bacteria shown in the charts. These subjects had bacterial taxa that were either absent or present only at substantially lower levels in many of the 36 subjects. *Moraxella* bacteria were found at high levels, particularly in nasal and nasopharyngeal samples. The percentage of OTUs that were not classified to genus level and shown as ‘other’ was 4.03, on average, with a standard deviation of 3.76, as calculated from the data for all 432 samples. Among these, 29 (6.71%) samples had more than 10% of ‘other’ OTUs, i.e., they could not be classified to the genus level by using the pipeline based on QIIME and the RDP classifier.

### Bacterial community diversity and variability

Within- and between-subject comparisons of microbiome diversity across regions and time points were conducted for 30 subjects that had a complete set of 24 swabs and at least 3000 reads per sample. Each of the 720 analyzed samples was rarified to 3000 reads.

**Fig 3:**
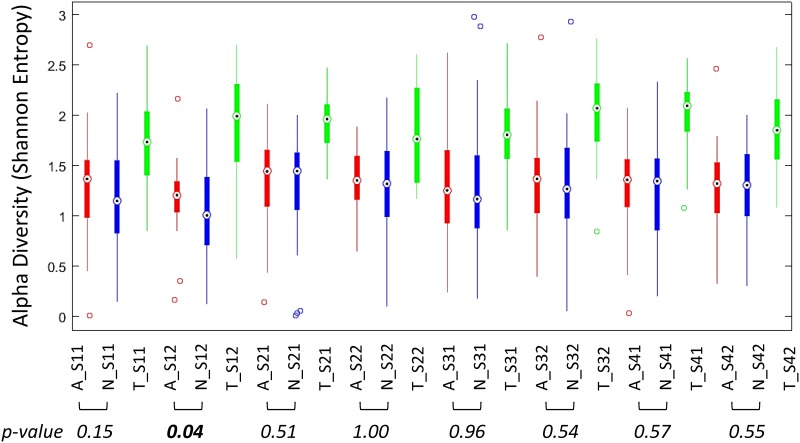
Variation in average α-diversity as a function of upper respiratory region and time. Within-subject α-diversity values for 30 subjects that had a complete set of 24 swabs and at least 3000 reads per sample were computed by using the Shannon entropy and averaged over each region and time point. Samples from the anterior nares (A) are red, samples from the nasopharynx (N) are blue, and samples from the oropharynx samples (T) are green. Plotting is performed using the ‘boxplot’ function with the standard (default) parameters. The filled bars (boxes) represent the 25^th^ (top) and 75^th^ (bottom) percentiles of the samples. The error bars cover the samples to the furthest distance within the error range. By default, an outlier is a value that is more than 1.5 times the interquartile range away from the top or bottom of the box. The diamond symbol represents the median of the samples.

Fig 3 summarizes the temporal variation of α-diversity for the three regions. The average α-diversity was higher in the oropharynx than in the other two regions, i.e., oropharyngeal communities were more diverse than in the other regions (Fig 3). A Wilcoxon signed-rank test for paired samples to compare the average microbiomes in the anterior nares and nasopharynx across the eight time points revealed no difference; only one of the eight tested sampled time points (S12, the second visit in the first quarter) showed a marginally significant difference (Fig 3). We used the same test to analyze the temporal variability of α-diversity in each region. The average α-diversity for the oropharyngeal community was larger than that for the other two regions at all time-points. We found no significant difference between the two visits regardless of the quarter (*P* > 0.05). For each of the two visits, we compared α-diversities between quarters for each of the three regions and found significant differences in 9 of the 36 comparisons (S2 Table). Although this suggests temporal variability of the respiratory microbiota for the studied military community, these results do not provide clear trends in temporal variability. The data could be analyzed by using additional methods. For example, the time dependence of individual respiratory microbiomes could be investigated [23]. In addition, the criteria to define extreme or unusual samples, subjects, or both need to be further explored.

**Fig 4:**
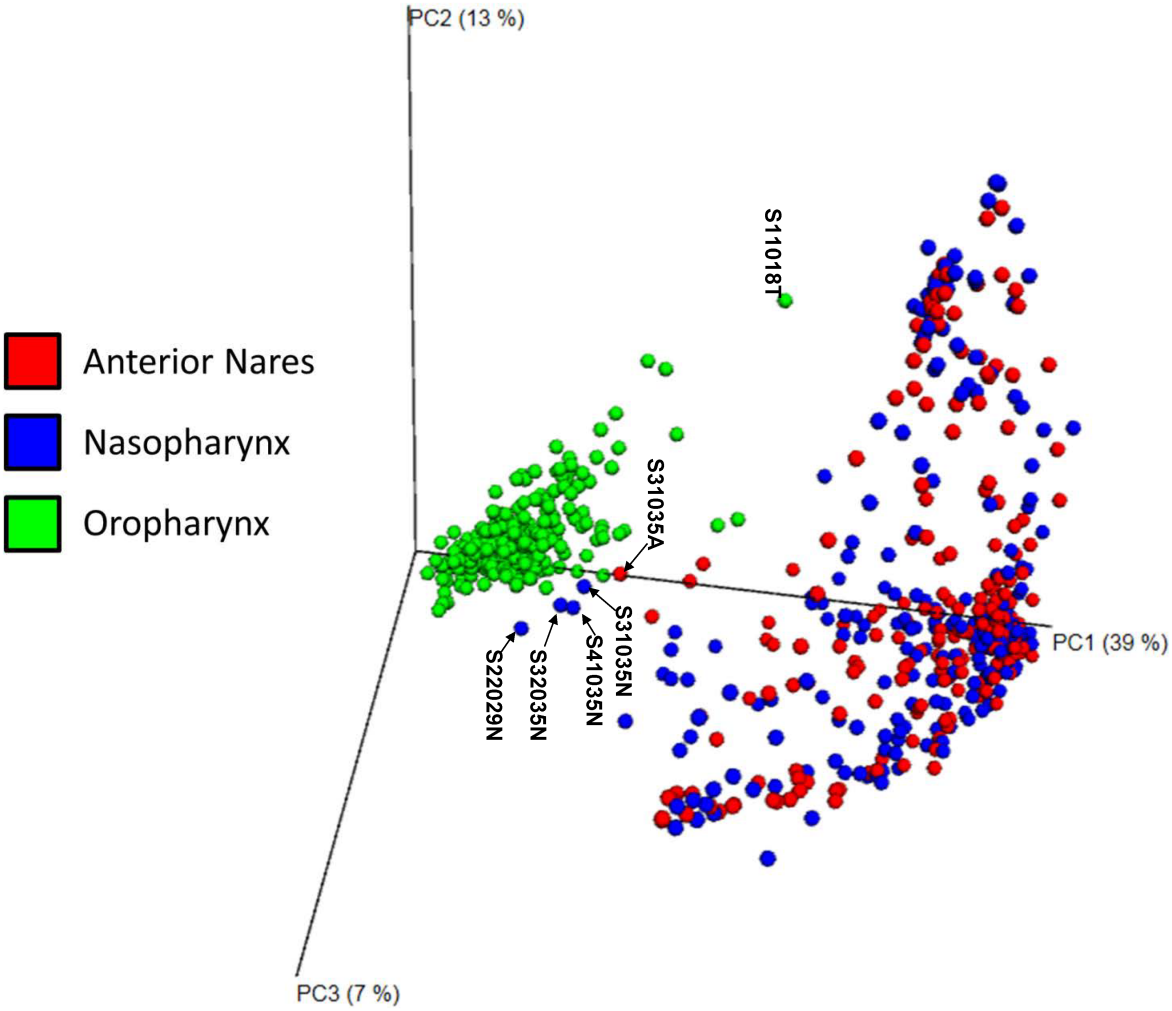
Variation in β-diversity as a function of upper respiratory regions. Between-subject β-diversity values were computed for 30 subjects that had a complete set of 24 swabs and at least 3000 reads per sample. The weighted UniFrac distance was used in the principal coordinate analysis of the distance matrices between the respiratory regions.

The inter-individual diversity (β-diversity) for 30 subjects (720 samples) was computed by using weighted UniFrac distances and visualized in Fig 4, using EMPeror [20]. The oropharyngeal region community formed a cluster of points well-separated from other two regions. There was significant overlap among anterior nares and nasopharyngeal samples. A few points “strayed” to the oropharyngeal cluster from the anterior nares and nasopharynx regions (Fig 4). These nasal and nasopharyngeal samples near the oropharyngeal cluster had a high content of *Streptococcus*, a dominant oropharyngeal genus [24–26]. Of the four samples from subject 35, three were from quarter S3 (Fig 4). In addition, one oropharyngeal sample was very far from the others (S11018T). Its compositional analysis indicated a high abundance of *Moraxella*, which was also observed in the anterior nares and nasopharyngeal samples of this subject. Abundant *Moraxella* in oropharyngeal samples of some subjects, including children and pneumonia patients, was also observed in other studies [6, 25–28].

Further study of the respiratory microbiomes of the healthy garrisoned military population in this project will provide a baseline understanding of normal respiratory microbiota, longitudinal within subject community composition stability and of variations in the population between individuals and across time. The large set of sequences for region V1–V3 of the bacterial 16S rDNA and the comprehensive demographic information for the 50 subjects contain valuable data that could be analyzed in depth, using specialized statistical tools, such as vegan (https://cran.r-project.org/web/packages/vegan) and metagenomeSeq [29], to determine the extent of association between the subject variables and microbiome profiles. Specific factors to be analyzed in the future include occupation (e.g., musician versus non-musician, wind instrument players versus other musicians), on-going use of asthma medications, influenza vaccination, and route of influenza vaccination (intramuscular versus intranasal).

## Supporting information

S1 Table Sample information and statistics of sequence reads. (XLSX)

S2 Table Temporal variability of alpha-diversity for three respiratory regions. (DOCX)

S1 Fig Bacterial community composition of respiratory microbiota in 36 human subjects. Bacterial taxa with an average relative abundance of ≥0.5% across the 36 samples are shown. Each sample name includes the quarter of collection 1–4 (*first digit*), visit 1 or 2 (*second digit*), subject number (*last three digits*), and type of swab (A, anterior nares; N, nasopharynx; T, oropharynx).
(XLSX)

## Abbreviations

16S rDNA: 16S ribosomal RNA gene
HMP: Human Microbiome Project
OTUs: operational taxonomic units
QIIME: Quantitative Insights Into Microbial Ecology
RDP: Ribosomal Database Project
rRNA: ribosomal RNA
