# Supplementary material for: Composition and variation of respiratory microbiota in healthy military personnel
Source: PLoS One. 2017 Dec 7;12(12):e0188461. doi: 10.1371/journal.pone.0188461 (PMC5720755; doi:10.1371/journal.pone.0188461)
Supplement: S2 Table — (DOCX) [file pone.0188461.s002.docx]

**S2 Table. Temporal variability of alpha diversity for three respiratory regions.** Comparisons were based on the Wilcoxon signed-rank test, using data from 30 subjects. *P* values of 0.05 or lower are shown in *bold*. Specimens were collected twice (V1 and V2) in each of the four specimen collection quarters, i.e., S1–S4. V1 data comparison results are shown in the *lower left* cells and *underlined*. V2 data comparison results are shown in the *upper right* cells.

|  | **Anterior Nares** | | | | **Oropharynx** | | | | **Nasopharynx** | | | |
| --- | --- | --- | --- | --- | --- | --- | --- | --- | --- | --- | --- | --- |
|  | S1 | S2 | S3 | S4 | S1 | S2 | S3 | S4 | S1 | S2 | S3 | S4 |
| S1 |  | **0.02** | **0.04** | 0.36 |  | 0.21 | 0.26 | 0.66 |  | **1E-04** | **0.04** | **0.01** |
| S2 | 0.92 |  | 0.32 | 0.9 | **0.006** |  | **0.05** | 0.48 | 0.46 |  | 0.73 | 0.72 |
| S3 | 0.81 | 0.32 |  | 0.95 | 0.17 | **0.05** |  | 0.17 | 0.72 | 0.97 |  | 0.81 |
| S4 | 0.72 | 0.9 | 0.95 |  | **0.01** | 0.48 | 0.17 |  | 0.27 | 0.9 | 0.75 |  |
